# Higher Age is Associated with Lower Likelihood of Conversion to Surgery after Primary Nonoperative Treatment for Osteochondral Lesions of the Talus

**DOI:** 10.1177/19476035241227357

**Published:** 2024-01-26

**Authors:** Tristan M.F. Buck, Jari Dahmen, J. Nienke Altink, Quinten G.H. Rikken, Inger N. Sierevelt, Sjoerd A.S. Stufkens, Gino M.M.J. Kerkhoffs

**Affiliations:** 1 Department of Orthopedic Surgery and Sports Medicine, Amsterdam UMC, Location AMC, University of Amsterdam, Amsterdam, The Netherlands; 2 Amsterdam Movement Sciences, Musculoskeletal Health, Amsterdam, The Netherlands; 3 Academic Center for Evidence-Based Sports Medicine (ACES), Amsterdam, The Netherlands; 4 Amsterdam Collaboration on Health & Safety in Sports (ACHSS), IOC Research Center, Amsterdam, The Netherlands; 5 Orthopedic Department, Spaarne Gasthuis Academy, Hoofddorp, The Netherlands; 6 Orthopedic Department, Xpert Clinics, Amsterdam, The Netherlands

**Keywords:** osteochondral lesions, talus, risk factors, hazard ratio, nonoperative and surgery

## Abstract

**Introduction:**

The first line of treatment for osteochondral lesions of the talus (OLT) is nonoperative. To date, there is limited evidence on risk factors that may influence conversion to surgery after primary nonoperative treatment for symptomatic OLTs. The aim of this study was therefore to identify risk factors for conversion to surgery after initial nonoperative treatment of OLTs.

**Methods:**

For this cohort study, patients with a primary OLT who were nonoperatively treated for at least 6 months between 1990 and 2020 were included. Univariable Cox regression analysis, resulting in hazard ratios (HRs), on the primary outcome (i.e. conversion to surgery after initial nonoperative treatment) was performed for potential risk factors. The following risk factors were analyzed: gender, age, body mass index (BMI), numeric rating scale (NRS), lesion size (depth, sagittal length, coronal length, volume, surface), lesion morphology (presence of fragments and presence of cysts), lesion location (medial/central/lateral), congruency of the ankle joint and trauma in history. Data imputation was conducted according to the multiple data principle with pooling.

**Results:**

Forty-two patients with primary OLTs were included in this study: 23 (55%) males and 19 (45%) females with a mean age of 39.1 (SD: 14.2). The median overall follow-up time was 66 months (range: 7-188). Around 23% of the patients had a conversion to surgery at the median observation time. The Kaplan-Meier analysis revealed a survival rate of 93% (95% confidence interval [CI]:84-100), 90% (95% CI: 81-99), and 77% (95% CI: 63-91) at 1, 2, and 5 years after the initiation of treatment, respectively. After performing the COX regression analysis, age was the sole risk factor significantly associated with conversion to surgery with an HR of 0.93 (95% CI: 0.87-0.99). The different HRs for all other risk factors were as follows: gender: 0.33 (95% CI: 0.08-1.34), BMI: 0.87 (95% CI 0.76-1.01), depth: 0.97 (95% CI: 0.79-1.18), coronal length: 1.19 (95% CI: 0.97-1.44), sagittal length: 0.98 (95% CI: 0.87-1.12), surface area: 1.17 (95% CI: 0.41-3.31), volume: 0.96 (95% CI: 0.24-3.91), presence of fragments: 4.17 (95% CI: 0.84-20.61).

**Conclusion:**

For primary OLTs, 77% of the patients were successfully treated nonoperatively at a median follow-up of 66 months without the need for a surgical intervention. Survival rates of 93%, 90%, and 77% were found at 1, 2, and 5 years after the initiation of treatment, respectively. We found that a higher age at the moment of diagnosis was significantly associated with a lower likelihood of conversion to surgery with a 7% decrease of likelihood each year the patient is older at the moment of diagnosis. The findings of this study are clinically relevant as it ameliorates the quality of the shared decision-making process between the patient and the treating team as we can advise OLT patients at a higher age with tolerable symptomatology that there is a relatively lower risk of conversion to surgery.

## Introduction

Treatment of symptomatic osteochondral lesions of the talus (OLTs) consists of a wide variety of treatment modalities.^1^ These strategies can be divided into nonoperative and surgical treatments.^2^ Prior to initiating surgical therapy, a nonoperative treatment approach can be chosen. Nonoperative treatment protocols consist of (a combination of) supervised neglect, restriction of high-loading activities, physical therapy, inlay soles, or weight reduction.^3,4^

A nonoperative treatment period should ideally be adhered to for at least 6 to 12 months.^2,3,5^ Patients with persisting or deteriorating symptoms regardless of nonoperative management may be considered candidates for surgical treatment, after appropriate counseling and a high-quality shared decision-making process.^1,2,6^

To date, several studies reported minimal amount of evidence regarding the success of nonoperative treatment.^3,4,7-9^ When studying the exact outcomes of these studies, it is clear that none have previously assessed the presence and magnitude of potential risk factors for conversion to surgery. These risk factors are important for clinical decision-making as they aid both the patient and the clinical team in increasing the quality of the (shared) decision-making process.

It is therefore the aim of this study to identify risk factors for conversion to surgery after initial nonoperative treatment for primary OLTs.

## Methods

The study was performed in accordance with the principles of the Declaration of Helsinki and the medical Research Involving Human Subjects Act (WMO) and with the ethical standards of our local institutional research committee. For this study, we received a waiver of the Medical Ethical Committee of the Amsterdam UMC, location AMC with reference number 08/326.

### Inclusion and Exclusion Criteria

Patients were eligible when treated nonoperatively for a primary osteochondral lesion of the talus in the Amsterdam UMC, location AMC, between the years of 1990 to 2019. The inclusion and exclusion criteria can be found in Table 1.

**Table 1.** Inclusion and Exclusion Criteria.

Inclusion criteria	Exclusion criteria
Primary OLT	Tibial lesions
Age: boys >16 years of age, girls >15 years of age	Symptomatic ankle instability
>6 months follow-up	Asymptomatic OLT

OLT = osteochondral lesion of the talus.

### Data Collection and Outcome

The primary outcome of this study was the association between the selected risk factors and the dichotomous outcome on conversion to surgery after initial nonoperative treatment (yes/no).

Secondary, overall success rates of conservative treatment were assessed at 1, 2, and 5 years follow-up.

Patients were included retrospectively from the electronic patient file system. All patient files were independently screened for baseline characteristics, risk factors and imaging characteristics by two researchers (TB, NA). Patients were additionally contacted through a telephone call in order to verify whether or not a surgical procedure had taken place in a different clinic.

### Risk Factors

The potential risk factors for conversion to surgery were based on clinical experience of two Foot and Ankle Fellowship-Trained experienced orthopedic surgeons (GK, SS), and review of the literature.^7,9,10^ These factors were: gender, age, body mass index (BMI), lesion size (depth, coronal length, sagittal length, surface and volume), morphology (cysts, presence of fragments), location (medial), congruency, corresponding alignment-location and ankle trauma (sprain, fracture) in the medical history, and Numeric Rating Scale (NRS) for pain during weight-bearing at baseline. The NRS for pain during weight-bearing at baseline is a scale ranging from 0 (*no pain*) to 10 (*worst pain imaginable*).^11^

### Imaging Outcomes

The following lesion characteristics were collected on computed tomography (CT) scans: baseline lesion size expressed in volume (cm^3^), surface (cm²), depth (mm), coronal length (mm), sagittal length (mm), presence of cysts (yes/no), presence of fragments (yes/no), and lesion location (medial/central/lateral). All these characteristics were reported by an attending experienced musculoskeletal radiologist and were registered in the electronic patient system. Another potential risk factor was hind-foot alignment as measured with anterior to posterior (AP) radiographs. Valgus and varus deformity were measured through the medial distal tibial angle (MDTA, normal values: 87°-91°).^12^ Valgus deformity was defined as MDTA > 91°, varus deformity was defined as MDTA < 87°. Valgus deformity can lead to higher joint load on the lateral part of the talar dome while a varus deformity may compress the medial part of the talar dome.^13^ Ankles with the combination of a medial OLT with varus alignment and a lateral OLT with valgus alignment were categorized as “corresponding alignment-location.” Ankles without these combinations were categorized as “noncorresponding.” “Corresponding alignment-location” was considered a risk factor in our analysis. Two independent researchers (TB, JA) measured the hindfoot alignment based on available weight-bearing radiographs to assess intraobserver and interobserver reliability. Congruency of the tibio-talar joint was determined by the difference between the tibial talar surface (TTS) and the tibial articular surface (TAS) angle (normal values 89.0° ± 2.3°).^12,14^ The joint was considered as congruent with a difference of less than 4° between TAS and TTS; a difference of 4° or more was considered as an incongruent joint.^15^

### Statistical Analysis

In case of normal distribution, continuous baseline characteristics were described through means and standard deviations, and in case of nonnormal distribution, these outcomes were described through medians and total range (min-max). Normality was checked by the use of the Shapiro-Wilkinson test. Categorical variables were described with absolute and relative frequencies. Owing to the importance of the “time to event,” a survival analysis was performed using the COX regression analysis. This analysis was used to identify the risk factors on conversion to surgery at each time point. The final observation date was set at 01-10-2021. After the univariable analysis, a multivariable analysis was planned with multiple risk factors that were considered statistically significant in the univariable analysis (*p* < .05).^16^ Results of the univariable analysis are expressed as hazard ratios (HR) with 95% confidence intervals (95% CI). Significance of the association between outcome and risk factors was calculated with the Wald statistics. Risk factors were considered statistically significant when the bilateral alpha risk was below 5%. An additional Kaplan-Meier curve is presented to calculate the overall success rate of nonoperative managed OLTs at 1, 2, and 5 years follow-up.

Missing data were considered as Missing Completely at Random and therefore, an imputation according to the multiple data principle with pooling could be conducted.^17^ Whether the data were Missing At Random (MAR), Not Missing At Random (NMAR), or Missing Completely At Random (MCAR) was determined based on missing patterns in the plots of missing data.

Interobserver and intraobserver agreement was assessed using the Intraclass Correlation Coefficient (ICC). A two-way random ICC model was used to calculate the interobserver agreement and a two-way mixed ICC model was used to calculate the intraobserver reliability. In addition, a Bland and Altman plot, including 95% levels of agreement (LOA), was used to assess the interobserver agreement and its measurement error. The ICC was interpreted as poor (0.40); moderate (0.40-0.75); substantial (0.75-0.90); or excellent reliability (>0.90).^18^

Statistical analyses were performed using R statistics software (version 3.6.3., 2020, R foundation for Statistical Computing, Vienna, Austria).

## Results

### Baseline Characteristics

Forty-two patients were included in this study. Baseline characteristics of the included patients and type of nonoperative treatment are summarized in Tables 2 and 3. Owing to missing data, not all patients are included in the calculation of rates in each type of nonoperative treatment.

**Table 2.** Baseline Characteristics, Demographic and Prior Treatments.

Demographics	
Age, (years)	
Mean ± SD	39.1 ± 14.2
Gender, number (%)	
Male	23 (55%)
Female	19 (45%)
Follow-up time in months, median (range)	66 (7-188)
Body mass index	
Mean ± SD	27.0 ± 5.7
Ankles, number (%)	
Right	18 (43%)
Left	24 (57%)
Type of nonoperative treatment (rate)	
PT	18/36
HA Injection	9/38
Inlay soles	21/37
Combinations of nonoperative treatment (n)	
Combination PT-HA Injection	5
Combination PT-Inlay soles	14
Combination HA injection-Inlay soles	7

HA = hyaluronic acid; PT = physical therapy.

**Table 3.** Baseline Characteristics, Lesion Characteristics.

Lesion Characteristics	
Lesion Size (Mean ± SD)	
Depth (mm)	7.4 ± 4.0
Coronal length (mm)	8.0 ± 3.3
Sagittal length (mm)	13.3 ± 5.5
Surface area (cm^2^)	0.94 ± 0.71
Volume (cm^3^)	0.55 ± 0.64
Morphological characteristics (rate)	
Cystic lesions	40/41
Presence of fragments	20/41
Location	
Medial/central/lateral	30/0/12
Medial distal tibial angle	
Mean (±SD)	89.9° ± 2.0°

### Treatment Outcomes

**Figure 1.**
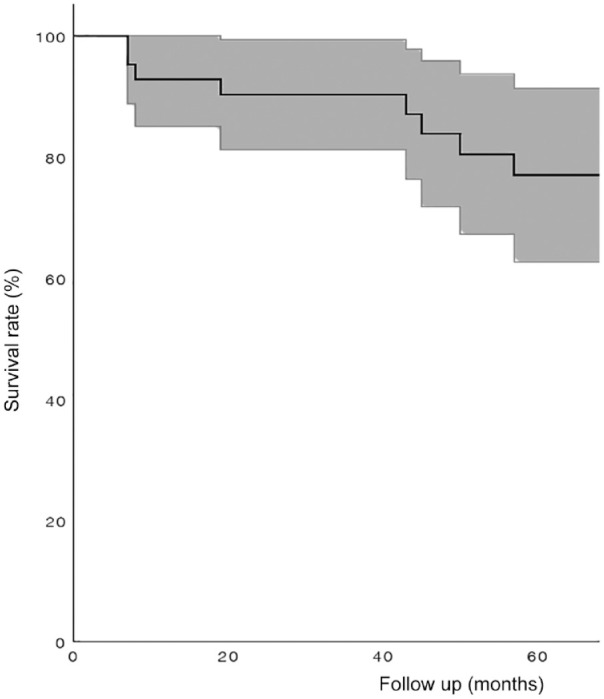
Kaplan-Meier curve for overall survival rate of initial nonoperative treated primary OLTs, including 95% CI. OLT = lesions of the talus; CI = confidence interval.

At a median follow up of 66 months (range: 7-188), nine patients (21%) needed a conversion to surgery after initial nonoperative treatment and 33 patients (79%) did not convert to surgery. The Kaplan-Meier analysis revealed a survival rate of 93% (95% CI: 84-100), 90% (95% CI: 81-99) and 77% (95% CI: 63-91) at 1, 2, and 5 years after the initiation of treatment, respectively (**Fig. 1**).

### Univariable Analysis

The univariable analysis showed a wide range in potential risk factors for conversion to surgery of which age was the only risk factor which was significantly associated with conversion to surgery (HR: 0.93, CI: 0.87-0.99, *P* < .05) (Table 4). Cystic morphology, congruency, corresponding alignment-location and trauma in history were not interpretable due to the fact that all patients in the group with conversion to surgery had a trauma in their medical history, a cystic lesion and a congruent ankle (Table 4). Therefore, the HRs were infinite and could not be interpreted. Corresponding alignment-location was not interpretable due to the lack of variation in the group that had conversion to surgery. None of the patients had a corresponding alignment-location.

**Table 4.** Risk Factors on Conversion to Surgery.

Risk Factor	HR	95% CI
Personal characteristics		
Gender: male	0.33	0.08-1.34
Age	0.93	0.87-0.99*
BMI	0.89	0.75-1.06
Lesion characteristics		
Depth	0.97	0.79-1.18
Coronal length	1.19	0.97-1.44
Sagittal length	0.98	0.86-1.11
Surface area (cm^2^)	1.18	0.42-3.31
Volume (cm^3^)	1.01	0.27-3.87
Cystic lesion	NA	NA
Presence of fragments	3.69	0.74-18.49
Radiology		
Location (medial)	1.80	0.36-9.27
Congruency	NA	NA
Corresponding alignment-location	NA	NA
Other		
Trauma in history	NA	NA

HR = hazard ratio; CI = confidence interval; BMI = body mass index; NA = not available due to the lack of variance.

* *P* < 0.05.

### Reliability of Imaging

**Figure 2.**
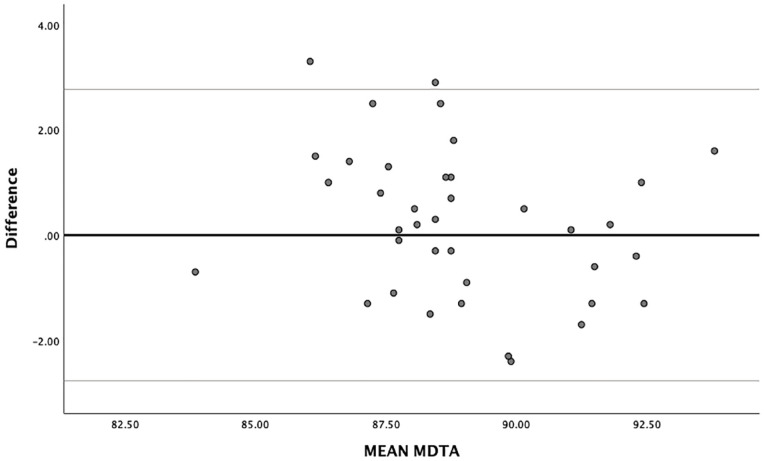
Bland and Altman plot with limits of agreement (LOA of the interobserver reliability). LOA = levels of agreement; MDTA = medial distal tibial angle.

The ICC for the interobserver reliability was 0.80 (substantial). The paired *t*-test showed no systematic differences between the two observers (p=0.33). The 95% limits of agreement were calculated as 2.77° and −2.77° (Fig. 2). For the intraobserver reliability, an ICC of 0.83 was also calculated being interpreted as substantial.^18,19^

### Missing Data

The following variables contained missing values: depth of the lesion (3 values), coronal diameter of the lesion (3 values), anterior-posterior diameter of the lesion (2 values), surface (4 values), volume (5 values), cystic change (1 value), presence of fragments (1 value), alignment and corresponding alignment-lesion nature (4 values). In addition, for 45% of our sample the BMI could be calculated. The missing data were considered as MCAR after concluding that missing values were not depending on values of either observed data or unobserved data. Data imputation was performed in accordance to the multiple data principle with pooling. The NRS for pain during weight-bearing at baseline was reported for two patients, thus making an analysis on this outcome in this study impossible.

## Discussion

The most important finding of this study was that a higher age was significantly associated with a lower risk of conversion to surgery after initial nonoperative treatment during a median follow-up of 66 months. In addition, although not significant, the presence of fragments and male gender showed a relative high risk of conversion to surgery.

Previous research and expert opinion studies have suggested that initial nonoperative therapy can be a clinically successful treatment for OLTs.^4,9,20^ More recently, a publication by Seo *et al.*,^9^ including 142 symptomatic OLT patients, studied the clinical outcomes of nonoperative therapy in the form of supervised neglect. They found that higher Berndt and Harty stages were associated with lower clinical outcome scores as assessed by visual analog scale (VAS) and the American Orthopedic Foot and Ankle Score (AOFAS). However, age, gender, and BMI were not associated with the AOFAS and VAS which is in contrast with our findings as these variables have, although not all significant, relatively high association with conversion to surgery.^9^

Our results showed 1, 2-, and 5-years success rates of 93% (95% CI: 84-100), 90% (95% CI: 81-99), and 77% (95% CI: 63-91), respectively. These decreasing rates of conservative success may be declared by the fact that lesions increase in size. Another factor that may play a role in this result is the progression of osteoarthritic changes which can cause deteriorating of symptoms. However, the lack of radiological follow-up made it impossible to draw hard conclusions on this. Future studies must focus on radiological follow-up in order to assess the radiologic changes in this specific group.

These high success rates in our study are in contrast with the findings of Tol *et al.*^4^ and Weiss *et al.*^21^ who found clinical effectiveness rates of 45% and 52%, respectively. The high success rates in our study and thus, the small number of patients that needed a conversion to surgery in this study could be explained by the fact that the study participants were treated in an academic clinical setting. Most of our patients came after referral by other hospitals, and already failed nonoperative treatment before they came to our clinic. It is imaginable that patients who were initially nonoperatively treated at our clinic have a lower level of symptoms and therefore a higher chance in succeeding on nonoperative management. In addition, the intensive guidance of a specialized team of physical therapists and orthopedic surgeons in the different types of nonoperative treatment, may play an important role in the succeeding of nonoperative management in our clinic. However, the influence of different types of supervised nonoperative management, such as HA injections, physical therapy, and inlay soles could not be investigated and need to be analyzed in further studies.

In this study, the factor age was a significant risk factor (*P* < .05), which was in line with previous findings.^21^ This could be explained by the fact that the older included people get, the lower their functional physical activity score.^20^ Thereby, older people may be satisfied with lower level of activity as a concession for less ankle complains. The presence of loose fragments showed a high HR for conversion to surgery, which is imaginable as instable loose fragments can cause symptoms such as locking and deep ankle pain. The finding that loose fragments, which can cause locking symptoms, could negatively influence the outcome of nonoperative management is comparable with prognostic findings for nonoperative management for osteochondral lesions of the knee.^22^

Trauma in history, presence of cysts and congruency showed uninterpretable results. The reason behind these results was the lack of variance in the collected data, which lead to an infinite HR and were therefore not interpretable. All surgically treated patients had congruent ankles, a trauma in history and cystic lesions. This lack of variance could be caused by the fact that our center is highly specialized in treating OLTs of the ankle.^20^ Therefore, patients mainly visit our center for having a personalized advice, whether surgical or nonoperative, after failed treatment in another hospital. They are mostly referred by other surgeons who already tried nonoperative treatment or small procedures like bone marrow stimulation. It is imaginable that patients who were initially treated nonoperatively in our center have a higher chance on succeeding nonoperative therapy due to possibly less pain in their ankle or a lower physical activity level. However, we could not confirm this with our data due to the lack of clinical outcome measurements at baseline. Despite the uninterpretable HRs for risk factors, our findings generate the hypothesis that these factors may play an important role in the survival-rate of nonoperative treatment.

This study needs to be interpreted in light of its strengths and limitations. Despite the low rate of conversion to surgery, this rate may not be regarded as a proper reflection of daily clinical care for patients with OLTs worldwide. First, referral bias is induced due to the tertiary character of our hospital which has an (inter)national status of an expert center in the treatment of OLTs. In addition, due to the retrospective character of this study, patients had a wide range in their observational time, which forced us to calculate HRs instead of odds ratios. Furthermore, during this study it was noticed that not all relevant risk factors were reported; initially, we considered NRS pain scores as a risk factor for potential conversion to surgery. However, this information had not been sufficiently reported in the electronic patient file and could therefore not be analyzed. In addition, this study consists only of univariable analysis, which ensures that results should be interpreted with caution. Moreover, the measurement of lesion size in all directions was extracted from the patient files, and there was no second rater for these risk factors. In future studies it is recommended to add an extra musculoskeletal radiologist to the research team to measure the size in order to improve the methodology. Firstly, a prospective methodological nature may be a more suitable manner to analyze predictive factors instead of risk factors. The study also has a number of strengths. This is the first study focusing on risk factors in conversion to surgery after initial nonoperative treatment. Furthermore, we regard the data imputation methods having been performed in our study a methodological strength as this completed data for other risk factors having been analyzed. It was expected that BMI was solely reported for patients with overweight and could therefore not be included in the imputation model as this would influence the results. However, in our data, it was seen that the rapportage of BMI had no tendency toward selectiveness for high body mass indexes (BMIs). Recommendations for further research on this topic consist of a prospective design for analyzing risk factors for conversion to surgery or for success of nonoperative therapy, including clinical outcome measurements at baseline and in the follow-up of these patients with standardized moments. This prospective study can also provide the ability to separate and analyze different modalities of nonoperative management, such as inlay soles or physical therapy, in a prospective manner. In addition, it is important to take psychological factors into account such as pain catastrophizing, coping strategies, and the influence of the injury on mental aspects of wellbeing. Furthermore, it is recommended that prospective research is conducted with a multicenter design in order to reduce or prevent referral bias.

The clinical 5 years success rate of 77% and the low risk of conversion to surgery are in line with previous findings and emphasize the important role of conservative treatment in the management of OLTs. Concerning the clinical relevance of the study, one can state that it was found that at the moment of diagnosis, a higher age was significantly associated with a lower likelihood of conversion to surgery with a 7% decrease each year the patient is older at diagnosis. Furthermore, these outcomes will help physicians in tailoring their management for individual patients.

## Conclusion

For primary OLTs, 77% of the patients were successfully treated nonoperatively at a median follow-up of 66 months without the need for a surgical intervention. Survival rates of 93%, 90%, and 77% were found at 1, 2, and 5 years after the initiation of treatment, respectively. We found that a higher age at the moment of diagnosis was significantly associated with a lower likelihood of conversion to surgery with a 7% decrease of likelihood each year the patient is older at the moment of diagnosis. The findings of this study are clinically relevant as it ameliorates the quality of the shared decision-making process between the patient and the treating team as we can advise OLT patients at a higher age with tolerable symptomatology that there is a relatively lower the risk of conversion to surgery.
